# SGLT2 Inhibitor Use in Chronic Kidney Disease: Supporting Cardiovascular, Kidney, and Metabolic Health

**DOI:** 10.1016/j.xkme.2024.100851

**Published:** 2024-06-08

**Authors:** Magdalena Madero, Glenn M. Chertow, Patrick B. Mark

**Affiliations:** a Department of Medicine, Division of Nephrology, Instituto Nacional de Cardiología—Ignacio Chávez, Mexico City, Mexico; b Departments of Medicine, Epidemiology and Population Health, and Health Policy, Stanford University School of Medicine, Stanford, CA; c School of Cardiovascular and Metabolic Health, University of Glasgow, Glasgow, UK

**Keywords:** Chronic kidney disease, sodium-glucose co-transporter-2 inhibitors, SGLT2 inhibitors, medication, prescribing

## Abstract

Originally developed for use in type 2 diabetes mellitus (T2DM), sodium–glucose co-transporter-2 (SGLT2) inhibitors demonstrated diverse cardiovascular- and kidney-protective effects in large outcome trials. Their subsequent approval as a treatment for chronic kidney disease (CKD) marked a pivotal shift in the landscape of CKD management. Further to this, the approval of dapagliflozin and empagliflozin for use in patients with CKD with and without T2DM afforded new treatment opportunities for this population. SGLT2 inhibitors provide an effective treatment for CKD with a favorable safety profile. However, their uptake has been slow, especially among patients without T2DM, owing perhaps to a lack of certainty and familiarity among health care professionals. As the landscape of CKD management continues to evolve, health care professionals should remain knowledgeable about these changes, and implement new guideline recommendations promptly to avoid therapeutic inertia. SGLT2 inhibitors are recommended for patients with CKD with or without T2DM and are foundational agents to support cardiovascular, kidney, and metabolic health. In this review, we provide evidence-based answers to questions that may be asked in the clinic regarding the use of SGLT2 inhibitors to treat CKD.

## SGLT2 Inhibitors: A Brief History

Sodium–glucose co-transporter-2 (SGLT2) inhibitors were developed to treat hyperglycemia in patients with type 2 diabetes mellitus (T2DM).^1^ However, in recent years, focus has shifted to their wide range of cardiovascular- and kidney-protective effects. Results from cardiovascular outcome trials showed that SGLT2 inhibitors have the potential to reduce chronic kidney disease (CKD) progression in patients with T2DM,^2^, ^3^, ^4^ with subsequent randomized controlled trials designed to investigate their safety and efficacy in patients with CKD (Table 1).^5^, ^6^, ^7^, ^8^

**Table 1:** Summary of Placebo-Controlled Trials of SGLT2 Inhibitors in Patients with CKD

Trial Name and Drug Studied	Patients, n	Median Follow-Up, y	eGFR, mL/min/1.73 m^2^	Median (IQR) UACR, mg/g	Key Eligibility Criteria	Primary Endpoint
CREDENCE^6^ (canagliflozin 100 mg)	4,401	2.6	Mean (SD): 56.3 (18.2)	923 (459-1,794)	Age ≥ 30 years; T2DM; eGFR ≥ 30 and < 90 mL/min/1.73 m^2^; UACR ≥ 300 and < 5,000 mg/g; stable dose of RAS inhibitor for ≥4 weeks before randomization	Composite of end-stage kidney disease (dialysis for ≥30 days, kidney transplantation, or eGFR < 15 mL/min/1.73 m^2^ for ≥30 days), doubling of serum creatinine levels from baseline sustained for ≥30 days, or death from renal or cardiovascular causes
DAPA-CKD^7^ (dapagliflozin 10 mg)	4,304	2.4	Mean (SD): 43.2 (12.3)	965 (472-1,903)	Age ≥ 18 years; eGFR ≥ 25 and < 75 mL/min/1.73 m^2^; UACR ≥ 200 and < 5,000 mg/g; stable dose of RAS inhibitor for ≥4 weeks before randomization^a^	Time-to-event analysis of the first occurrence of any of the following: ≥50% decline in eGFR from baseline, end-stage kidney disease (dialysis for ≥28 days, kidney transplantation, or eGFR < 15 mL/min/1.73 m^2^ for ≥28 days), or death from renal or cardiovascular causes
SCORED^5^ (sotagliflozin 200-400 mg)	10,584	1.3	Median (IQR): 44.5 (37.0-51.4)	74 (17-481)	Age ≥ 18 years; T2DM; eGFR ≥ 25 and ≤ 60 mL/min/1.73 m^2^	Total number of deaths from cardiovascular causes, hospitalizations for heart failure, and urgent visits for heart failure^b^
EMPA-KIDNEY^8^ (empagliflozin 10 mg)	6,609	2.0	Mean (SD): 37.3 (14.5)	329^c^	Age ≥ 18 years; eGFR ≥ 20 and < 45 mL/min/1.73 m^2^ (regardless of UACR) or eGFR ≥ 45 and < 90 mL/min/1.73 m^2^ with UACR ≥ 200 mg/g; stable dose of a single RAS inhibitor	First occurrence of progression of kidney disease (end-stage kidney disease [initiation of maintenance dialysis or kidney transplantation], sustained eGFR of <10 mL/min/1.73 m^2^, sustained decrease from baseline eGFR of ≥40%, or death from renal causes) or death from cardiovascular causes

Abbreviations: CKD, chronic kidney disease; eGFR, estimated glomerular filtration rate; IQR, interquartile range; RAS, renin–angiotensin system; SD, standard deviation; SGLT2, sodium–glucose co-transporter-2; T2DM, type 2 diabetes mellitus; UACR, urinary albumin-creatinine ratio.

a Patients documented as being unable to take RAS inhibitors were also allowed to participate in DAPA-CKD.

b The original coprimary endpoints for the SCORED trial were time-to-event of first occurrence of a major cardiovascular event (death from cardiovascular causes, nonfatal myocardial infarction, or nonfatal stroke) and the first occurrence of death from cardiovascular causes or hospitalization for heart failure. The SCORED trial closed early because of lack of funding, leading to the primary endpoint being changed.

c IQR for UACR in all patients in the EMPA-KIDNEY trial were not reported, median (IQR) UACR was 331 mg/g (46-1,061) and 327 mg/g (54-1,074) in the empagliflozin and placebo groups, respectively.

Results from these trials led to the approval of SGLT2 inhibitors to treat CKD. In 2020, canagliflozin was approved by the US Food and Drug Administration (FDA) to reduce the risk of end-stage kidney disease, doubling of serum creatinine levels, cardiovascular death, and hospitalization in adults with T2DM and diabetic kidney disease with albuminuria. Initiation of canagliflozin is indicated in patients with an estimated glomerular filtration rate (eGFR) > 30 mL/min/1.73 m^2^ (treatment may be continued in patients whose eGFR decreases to below this threshold).^9^ In 2021, dapagliflozin was approved by the FDA to reduce the risk of sustained eGFR decline, end-stage kidney disease, cardiovascular death, and hospitalization for heart failure in adults with CKD at risk of progression, regardless of T2DM status. Initiation of dapagliflozin is indicated in patients with an eGFR > 25 mL/min/1.73 m^2^ (treatment may be continued if their eGFR decreases to below this threshold).^10^ In 2023, empagliflozin received FDA approval to reduce the risk of sustained eGFR decline, end-stage kidney disease, cardiovascular death, and hospitalization in adults with CKD at risk of progression, regardless of T2DM status.^11^^,^^12^ The FDA prescribing information notes that efficacy and safety trials of empagliflozin did not enroll adult patients with eGFR < 20 mL/min/1.73 m^2^,^11^ and the European Union prescribing information does not recommend initiation of empagliflozin in patients with an eGFR < 20 mL/min/1.73 m^2^.^13^

Although T2DM is highly prevalent among patients with CKD, a substantial proportion of patients with CKD do not have T2DM.^14^^,^^15^ The approval of dapagliflozin and empagliflozin to treat CKD irrespective of T2DM status has added a new option for disease management in this population. After 2 decades of renin–angiotensin system (RAS) inhibitors being the mainstay of CKD treatment, health care professionals now have new and highly effective tools to manage CKD in patients with and without T2DM. SGLT2 inhibitors are now recommended as cofirst-line therapy alongside RAS inhibitors in the majority of patients with CKD.^16^, ^17^, ^18^ Some patient populations were not included in pivotal trials, including patients with polycystic kidney disease, lupus nephritis, antinuclear cytoplasmic antibody (ANCA)-associated vasculitis, and those with a history of kidney transplantation, and as such, recommendations in these populations can vary. SGLT2 inhibitors are no longer solely drugs for diabetes—their kidney-protective effects have now firmly established them as kidney medicines.

## SGLT2 Inhibitors for CKD: From Trial Results to Clinical Practice

Despite the broadening range of evidence supporting the use of SGLT2 inhibitors, their uptake to treat CKD in clinical practice has been slow,^19^ particularly in patients without T2DM.^20^ This high degree of therapeutic inertia exposes patients to an undue risk of CKD progression and adverse outcomes,^21^^,^^22^ as well as resulting in a substantial economic burden to health care systems.^23^, ^24^, ^25^

Evidence-based clinical practice guidelines continue to evolve in response to new data.^26^^,^^27^ Increasing focus is being placed on early diagnosis and treatment of CKD,^28^ and SGLT2 inhibitors provide an effective option with a good safety profile for disease management. In addition to effects on slowing CKD progression, SGLT2 inhibitors have been shown to reduce the risk of acute kidney injury, cardiovascular death, and hospitalization for heart failure.^29^ Additionally, owing to the high cost of renal replacement therapy, results from clinical trials indicate that SGLT2 inhibitors are cost-effective in patients with CKD,^30^^,^^31^ with and without T2DM.^32^

**Figure 1:**
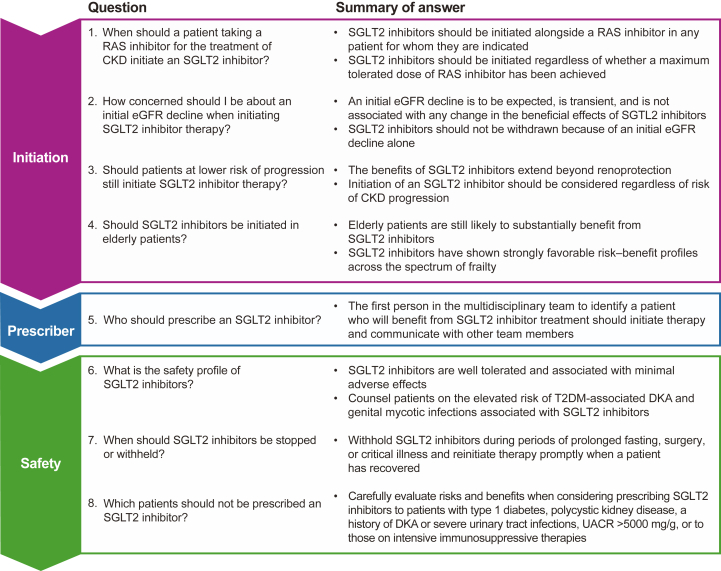
Summary of questions and answers. Abbreviations: CKD, chronic kidney disease; DKA, diabetic ketoacidosis; eGFR, estimated glomerular filtration rate; RAS, renin–angiotensin system; SGLT2, sodium–glucose co-transporter-2; T2DM, type 2 diabetes mellitus; UACR, urinary albumin-creatinine ratio.

As treatment landscapes continue to shift, it is important that health care professionals adapt to take full advantage of the benefits that SGLT2 inhibitors provide. This article aims to reinforce guidelines by providing evidence-based answers to specific questions regarding SGLT2 inhibitor use in patients with CKD (Figure 1).

## Question: When Should a Patient Taking a RAS Inhibitor for the Treatment of CKD Initiate an SGLT2 Inhibitor?

Guidelines recommend that SGLT2 inhibitors are prescribed alongside RAS inhibitors for the treatment of CKD^27^ given the strong kidney-protective benefits offered by this combination of therapies.^33^, ^34^, ^35^ Although we support the practice of uptitration of RAS inhibitors, uptitration of RAS inhibitors should not lead to undue delay in initiating SGLT2 inhibitors. This is particularly important given the observed benefits of SGLT2 inhibitors across a range of RAS inhibitor doses, and the early separation of cumulative incidence curves in clinical trials, particularly for the composite cardiovascular endpoint (hospitalization for heart failure or cardiovascular death).^29^ A prespecified analysis of the Dapagliflozin and Prevention of Adverse Outcomes in Chronic Kidney Disease (DAPA-CKD) trial found that dapagliflozin was effective at reducing the risk of the primary and secondary endpoints (time to composite kidney endpoint of ≥50% sustained eGFR decline, kidney failure, or death from kidney disease; time to composite cardiovascular endpoint of hospitalization for heart failure or cardiovascular death; time to death from any cause) versus placebo irrespective of RAS inhibitor dosage.^36^ The DAPA-CKD trial also showed that dapagliflozin reduced all-cause mortality,^37^ a benefit not observed with RAS inhibition alone.^38^ As such, there is no need to delay initiation of SGLT2 inhibitors until after a maximum tolerated dose of RAS inhibitor is achieved given that the benefit does not appear to be dependent on RAS inhibitor dose.^36^

It is often necessary to uptitrate the dose of RAS inhibitors to achieve the desired treatment effect while avoiding hyperkalemia.^39^ Doing so necessitates additional monitoring of serum creatinine and potassium concentrations, which is a time-consuming process for patients and health care professionals. On the contrary, SGLT2 inhibitors do not require such additional monitoring after initiation. Unlike RAS inhibitors,^40^ SGLT2 inhibitors are not associated with an elevated risk of hyperkalemia.^41^^,^^42^ A meta-analysis of clinical trials found that SGLT2 inhibitors reduce the risk of hyperkalemia in patients with T2DM at high cardiovascular risk or with CKD, without increasing the risk of hypokalemia.^43^

Approximately 1,000 patients enrolled in EMPA-KIDNEY received empagliflozin as monotherapy, without RAS inhibitors.^8^ SGLT2 inhibitor monotherapy should be considered as an option in patients who are intolerant to RAS inhibitors or for whom RAS inhibitors are contraindicated.

## Question: How Concerned Should I Be About an Initial eGFR Decline When Initiating SGLT2 Inhibitor Therapy?

Owing to their renal mechanism of action, patients who initiate an SGLT2 inhibitor may experience a decline in eGFR shortly after beginning treatment,^44^ similar to that observed in some patients after initiating RAS inhibitors.^45^

An initial decline in eGFR is to be expected and should not be considered a cause for concern. A post hoc analysis of the EMPA-REG OUTCOME trial results found that an initial eGFR decline from baseline of >10% occurred more often in patients treated with empagliflozin than in those receiving placebo (28.3% vs 13.4% of patients). This eGFR decline did not modify the beneficial cardiovascular or kidney effects of empagliflozin, and mean eGFR remained stable from week 12 onward.^44^ A post hoc analysis of the CREDENCE trial results also found that although a transient eGFR decline (>10% from baseline) occurred more often in the canagliflozin arm than in the placebo arm (44.6% vs 21.0% of patients), the clinical benefit of canagliflozin was still observed.^46^ Similarly, a prespecified analysis of the DAPA-CKD trial showed that an eGFR decline (>10% from baseline) 2 weeks after initiation of dapagliflozin was a transient event and was not associated with CKD progression or changes in clinical benefits or outcomes.^47^ Monitoring of eGFR after SGLT2 inhibitor initiation is not necessary. However, Kidney Disease: Improving Global Outcomes (KDIGO) guidelines recommend that a significant reduction of >30% in eGFR following initiation of an SGLT2 inhibitor warrants further investigation and monitoring.^26^ An initial eGFR decline alone should not lead to withdrawal of SGLT2 inhibitors because these effects are typically reversible and not indicative of drug toxicity.^48^

## Question: Should Patients at Lower Risk of Progression Still Initiate an SGLT2 Inhibitor?

Health care professionals may be hesitant to prescribe SGLT2 inhibitors to patients with CKD at a lower risk of progression, such as patients with albuminuria and preserved eGFR or those with impaired kidney function and low urinary albumin-creatinine ratio (UACR), owing to limited evidence from clinical trials in these patient groups. Although the EMPA-KIDNEY trial showed that empagliflozin was not associated with a significant reduction in the risk of primary outcome in patients with UACR ≤ 30 mg/g, there was evidence of a favorable effect on eGFR slope in this population.^8^ Additionally, the 3 major trials and a meta-analysis showed that there were benefits of SGLT2 inhibitor therapy across the range of eGFRs and albuminuria without substantial heterogeneity of effect.^29^

Recently published guidelines from the European Society of Hypertension recommend the use of SGLT2 inhibitors in addition to lifestyle interventions in patients with CKD and hypertension.^17^ Furthermore, alongside a class 1A recommendation to use SGLT2 inhibitors to treat CKD in patients with eGFR ≥ 20 mL/min/1.73 m^2^ and a UACR ≥ 200 mg/g, the newly updated KDIGO guidelines also suggest using SGLT2 inhibitors in patients with eGFR ≥ 20 to 45 mL/min/1.73 m^2^ and a UACR < 200 mg/g as a class 2B recommendation.^18^ This suggestion is largely based on findings from the EMPA-KIDNEY trial and other trials in which benefits were observed among patients without macroalbuminuria^8^; results from real-world studies suggest that the benefits of SGLT2 inhibitors are preserved among patients with lower levels of albuminuria.^49^^,^^50^

**Figure 2:**
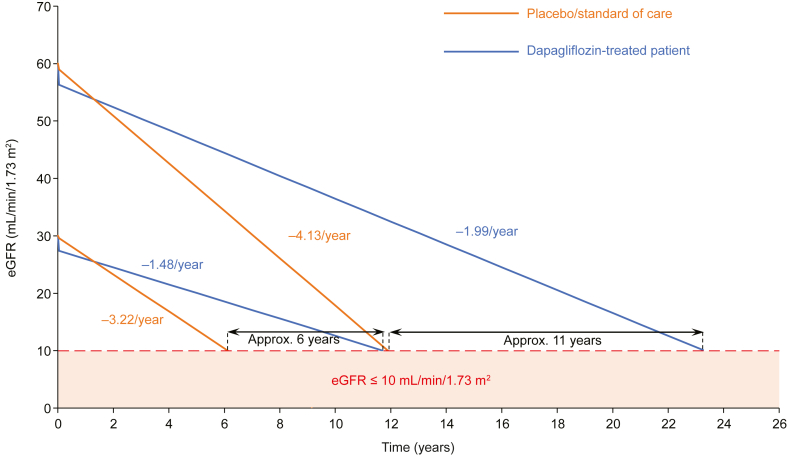
Extrapolation of data from the placebo-controlled DAPA-CKD clinical trial suggests that initiation of SGLT2 inhibitors at a higher eGFR could delay kidney failure by a longer time than initiation at a lower eGFR.^51^ Initial eGFR decline over the first 2 weeks was estimated using eGFR slope data from the acute phase of the DAPA-CKD trial. Projections are based on the eGFR from week 2 to end of treatment (DAPA-CKD chronic phase) and assume linear progression of eGFR decline. Example patient with initial eGFR of 60 mL/min/1.73 m^2^ used eGFR slope data from the DAPA-CKD trial subgroup with eGFR ≥ 45 mL/min/1.73 m^2^; eGFR at the start of the chronic phase: dapagliflozin 10 mg, 56.3 mL/min/1.73 m^2^; placebo, 59.0 mL/min/1.73 m^2^. Example patient with initial eGFR of 30 mL/min/1.73 m^2^ used eGFR slope data from the DAPA-CKD trial subgroup with eGFR < 45 mL/min/1.73 m^2^; eGFR at the start of the chronic phase: dapagliflozin 10 mg, 27.4 mL/min/1.73 m^2^; placebo, 29.6 mL/min/1.73 m^2^. Abbreviations: CKD, chronic kidney disease; eGFR, estimated glomerular filtration rate; SGLT2, sodium–glucose co-transporter-2.

Given their ability to preserve eGFR, initiation of an SGLT2 inhibitor is likely to significantly delay progression to kidney failure. A post hoc analysis of results from the DAPA-CKD trial found that dapagliflozin significantly slowed the rate of eGFR decline in patients with CKD versus placebo, regardless of T2DM status.^51^ Extrapolating data from this analysis highlights the potential benefits of early SGLT2 inhibitor initiation, suggesting that treatment initiation (vs no initiation) when a patient has an eGFR of 60 mL/min/1.73 m^2^ could potentially delay kidney failure by approximately 11 years, whereas initiation at an eGFR of 30 mL/min/1.73 m^2^ could delay kidney failure by approximately 6 years (Figure 2).^51^ It is important to bear in mind the assumptions made when extrapolating trial data in this way. Specifically, these estimates do not take into account competing risk of death, and they assume that adherence is maintained, that benefits are sustained over time, and that there are no longer-term adverse effects. However, the benefit of delaying end-stage kidney disease should not be understated, and if patients stand to benefit from an SGLT2 inhibitor, it should be considered regardless of their risk of CKD progression.

## Question: Should SGLT2 Inhibitors Be Initiated in Elderly Patients?

The prevalence of CKD tends to increase with age,^52^ owing to the increased prevalence of CKD risk factors such as T2DM, hypertension, and cardiovascular disease.^53^ Therefore, although eGFR does decline with age,^54^ it is still important to consider CKD as a distinct medical condition and treat appropriately.

Use of SGLT2 inhibitors for CKD treatment is not contraindicated in the elderly. Prescribers may, however, be concerned that patient populations in clinical trials of SGLT2 inhibitors were not representative of patients aged >65 years (in the CREDENCE, DAPA-CKD, and EMPA-KIDNEY trials, the mean age of patients treated with SGLT2 inhibitors was 63 years, 62 years, and 64 years, respectively).^3^^,^^7^^,^^8^ A prespecified analysis of data from the DAPA-CKD trial found that dapagliflozin was associated with a significant benefit on eGFR slope versus placebo in patients aged >65 years.^51^ Furthermore, a prospective observational study of patients aged ≥75 years hospitalized for acute decompensated heart failure with stage 3-4 CKD found that SGLT2 inhibitors were associated with slower eGFR decline than loop diuretics.^55^ In addition, recently published post hoc analyses of data from the CREDENCE and DAPA-CKD trials showed a consistent reduction in the risk of kidney events in patients across all age groups.^56^^,^^57^ In these analyses, the absolute risk of the kidney composite endpoint was lowest in patients aged ≥70 years and ≥80 years in the CREDENCE and DAPA-CKD trials, respectively.^56^^,^^57^ Moreover, the benefits of SGLT2 inhibitors on reducing the risk of hospitalization for heart failure or cardiovascular death are particularly relevant for elderly patients with (and without) CKD.^58^ Advanced age should not be seen as a barrier to treatment; elderly patients are still likely to experience substantial benefits from SGLT2 inhibitors.

Prescribers may be concerned about the need to minimize polypharmacy in patients with various comorbid conditions. A prespecified analysis of the DAPA-CKD trial found that there was no increase in serious adverse events when dapagliflozin was used in combination with a diuretic, RAS inhibitor, β-adrenergic antagonist, or calcium channel blocker, or several of these drugs in combination.^36^

Frailty may also be a concern in the elderly. However, a recent subanalysis of results from the DAPA-CKD trial showed that the effects of dapagliflozin on reducing the risk of primary and secondary outcomes versus placebo were maintained regardless of patient frailty.^59^ In addition, the frequency of serious adverse events was lower in patients randomized to dapagliflozin (vs placebo) regardless of frailty.^59^ Together, the results of this analysis suggest a strongly favorable risk–benefit profile for the use of dapagliflozin in patients with CKD across the spectrum of frailty.

## Question: Who Should Prescribe an SGLT2 Inhibitor?

Different health care professionals are likely to see patients at different stages of CKD. Patients with early-stage CKD are likely to be encountered in primary care, whereas patients with more advanced CKD are frequently managed either with, or primarily by, nephrologists. Primary care providers are no doubt familiar with SGLT2 inhibitors from extensive experience with T2DM; however, SGLT2 inhibitors are now indicated to treat CKD in patients with and without T2DM.^9^, ^10^, ^11^

The first person in the multidisciplinary team to identify patients likely to benefit from SGLT2 inhibitors should initiate treatment promptly and communicate with other team members. This could be a primary care physician or nonphysician primary care provider, nephrologist, endocrinologist, or cardiologist. No matter who writes the prescription, commencing therapy with an SGLT2 inhibitor for a patient who is apt to benefit should not be delayed. The effect of SGLT2 inhibitors (vs placebo) on outcomes in the CREDENCE, DAPA-CKD, and EMPA-KIDNEY trials tended to be seen within several months of starting treatment.^6^, ^7^, ^8^ Years-long delays in SGLT2 inhibitor prescription for patients who would benefit from initiating these drugs should therefore be strongly discouraged.

One can readily understand why there might have been reluctance to prescribe SGLT2 inhibitors in the primary care setting. When these agents were initially approved for use as glucose-lowering drugs, it was apparent that efficacy in improving glycemic control was attenuated with lower levels of kidney function.^60^ As a result, SGLT2 inhibitors were initially not recommended for patients with eGFR < 45 mL/min/1.73 m^2^ (canagliflozin and empagliflozin)^61^^,^^62^ or 60 mL/min/1.73 m^2^ (dapagliflozin).^63^ Furthermore, when the first wave of clinical trials was conducted in patients with T2DM, it was assumed that the observed cardiovascular benefits were linked to improved glycemic control. It was only later that we learned that the cardiovascular and kidney benefits of SGLT2 inhibitors were largely independent of glycemic control.^64^ Moreover, on the basis of early clinical experience, there was concern for an increased risk of acute kidney injury following exposure to SGLT2 inhibitors.^65^ More clinical experience and multiple clinical trials (including those that enrolled patients with CKD) have highlighted the fact that patients treated with SGLT2 inhibitors are significantly less likely to experience acute kidney injury than patients treated with placebo.^29^

## Question: What Is the Safety Profile of SGLT2 Inhibitors?

As with any medicine, it is important to consider the potential benefits and harms for patients before prescribing. SGLT2 inhibitors are well tolerated and associated with a reduction in the risk of kidney disease progression, acute kidney injury, cardiovascular death, or hospitalization for heart failure in patients with or without T2DM, with benefits from all trial populations studied to date considerably outweighing the risk of serious adverse events.^29^

Prescribers should still be aware of potential adverse effects and counsel patients appropriately. For example, SGLT2 inhibitors are associated with an increase in the risk of genital mycotic infections,^66^ with the highest incidence observed in women, patients with a history of these infections, and patients with poor glycemic control,^67^ for whom glycosuria is most prominent. The risk of genital mycotic infections in patients without T2DM is low, particularly those with lower eGFR, for whom the degree of glycosuria is diminished.^68^ The risk of genital mycotic infections can be managed by counseling patients on genital hygiene (eg, bathing soon after exercise, bathing at night, and drying off completely before dressing if bathing in the morning). Genital mycotic infections associated with SGLT2 inhibitor use are typically mild, and uncomplicated infections can be treated with oral or topical antifungals.^69^^,^^70^

SGLT2 inhibitors have been associated with an increased risk of euglycemic diabetic ketoacidosis (DKA).^71^^,^^72^ An observational study of patients in the United States identified several risk factors for hospitalization owing to SGLT2 inhibitor-associated DKA, including use of digoxin or medication for dementia, history of intracranial hemorrhage or history of DKA, recent hypoglycemia, high baseline glycated hemoglobin (>10%), and low baseline bicarbonate (<18 mmol/L).^73^ Other suggested risk factors for SGLT2 inhibitor-associated DKA include reduction in insulin dose, surgical stress, caloric restriction, alcohol abuse, and latent autoimmune diabetes in adults.^74^ Although the absolute risk of SGLT2 inhibitor-associated DKA in patients with T2DM is low,^29^ patients should still be counseled on the risk of DKA and advised to seek medical care for any signs or symptoms. The risk of ketoacidosis among patients without T2DM is very low; a meta-analysis of SGLT2 inhibitor clinical trials found only 1 event of ketoacidosis in such patients receiving an SGLT2 inhibitor during approximately 30,000 patient-years of follow-up.^29^ SGLT2 inhibitor-associated DKA should therefore not be considered a barrier to prescribing in patients without T2DM.

Initial safety concerns regarding elevated risk of lower limb amputation associated with SGLT2 inhibitors arose from early clinical trials. Results from the CANVAS and CANVAS-R trials showed that canagliflozin was associated with an increase in the occurrence of lower limb amputation, with the highest absolute risk in patients with pre-existing peripheral vascular disease, history of amputation, neuropathy, and increased risk of infection.^75^^,^^76^ However, no significant association between lower limb amputation and treatment with other SGLT2 inhibitors, including dapagliflozin or empagliflozin, was identified in clinical trials.^7^^,^^8^^,^^77^^,^^78^ In a meta-analysis of 13 SGLT2 inhibitor trials, the risk of lower limb amputation was low among patients allocated to an SGLT2 inhibitor, accounting for 1 lower limb amputation event per 1,000 patient-years in patients with T2DM.^29^ This meta-analysis also found that the risk of lower limb amputation was markedly lower in patients without diabetes than in those with diabetes.^29^ Given that the occurrence of this adverse effect is rare, the benefits of SGLT2 inhibitors far outweigh this risk, particularly among patients without T2DM. Furthermore, if lower limb amputation is a concern, risk can be mitigated through careful monitoring by practitioners and routine preventative foot care.

## Question: When Should SGLT2 Inhibitors Be Stopped or Withheld?

During times of prolonged fasting, surgery, or critical medical illness, when the risk of ketoacidosis is heightened, SGLT2 inhibitors should be withheld. Guidance from the FDA, updated in 2022, recommends stopping SGLT2 inhibitors at least 3 days before surgery.^79^ Patients should also be counseled to stop taking an SGLT2 inhibitor if they are unable to eat or drink, for example because of severe nausea or vomiting or prolonged periods of religious fasting, again because of elevated risk of DKA. SGLT2 inhibitors should be reinitiated promptly when the risk of DKA has normalized.

Initiation of SGLT2 inhibitors is not recommended in patients with an eGFR < 20 mL/min/1.73 m^2^.^9^, ^10^, ^11^^,^^18^ However, owing to the wide range of benefits associated with SGLT2 inhibitors, they should not be withdrawn if a patient’s eGFR decreases to below 20 mL/min/1.73 m^2^, unless they are not tolerated or a patient begins renal replacement therapy.^18^ Although the safety and efficacy of SGLT2 inhibitors have not been formally established in patients with eGFR < 20 mL/min/1.73 m^2^, patients enrolled in the CREDENCE, DAPA-CKD, and EMPA-KIDNEY trials were receiving therapy while their kidney function declined (albeit at slower rates than patients on placebo),^6^, ^7^, ^8^ sometimes after the initiation of dialysis.^80^ It should also be noted that current studies are in progress to investigate the use of SGLT2 inhibitors in patients undergoing kidney replacement therapy, for example the RENAL LIFECYCLE trial (NCT05374291).^81^

## Question: Which Patients Should Not Be Prescribed an SGLT2 Inhibitor?

Readers should be reminded that patients with polycystic kidney disease were excluded from participation in the 3 major CKD-focused trials, as were patients with UACR > 5,000 mg/g and patients receiving intensive immunosuppressive therapy (including kidney and other solid organ transplant recipients). However, these should not be considered absolute contraindications to use of SGLT2 inhibitors; rather, anticipated risks and benefits should be considered on an individual basis. As the American astronomer Carl Sagan stated, “The absence of evidence is not evidence of absence.” A trial funded by the National Institutes of Health is investigating the potential benefit of SGLT2 inhibitors in patients with autosomal dominant polycystic kidney disease, with the results expected in 2025.^82^ Owing to the risk of DKA, it is not recommended to prescribe SGLT2 inhibitors to patients with type 1 diabetes or a history of DKA.^26^ However, as outlined above, appropriate risk factor management and patient counseling can help to mitigate the risk of serious SGLT2 inhibitor-associated DKA.

Severe urinary tract infections have been reported in patients with obstructive conditions and/or with urinary diversion and chronic bacterial colonization; therefore, caution should be taken when prescribing SGLT2 inhibitors to these patients.^83^

## Conclusions

The development of SGLT2 inhibitors and their subsequent approval for the treatment of CKD in a wide range of patient populations represents a landmark shift in the management of the disease. In particular, the approval of dapagliflozin and empagliflozin for use in patients with CKD without T2DM offers this patient population an effective option to manage their condition, typically in conjunction with RAS inhibitors, but also in patients in whom RAS inhibitors are contraindicated because of hyperkalemia or hypotension. Given the wide range of benefits associated with the use of SGLT2 inhibitors, primary care and specialty care providers should consider using these agents in appropriate populations without delay.
